# Supplementary material for: High-performance ferroelectric field-effect transistors with ultra-thin indium tin oxide channels for flexible and transparent electronics
Source: Nat Commun. 2024 Mar 27;15:2686. doi: 10.1038/s41467-024-46878-5 (PMC10973520; doi:10.1038/s41467-024-46878-5)
Supplement: Supplementary file 1 — Supplementary Information [file 41467_2024_46878_MOESM1_ESM.pdf]

Supplementary Information for

High-performance ferroelectric field-effect transistors with ultra-thin indium tin oxide channels for flexible and transparent electronics

Qingxuan Li[1, 2, # *], Siwei Wang[2, #], Zhenhai Li[2], Xuemeng Hu[2], Yongkai Liu[2], Jiajie Yu[2], Yafen Yang[2], Tianyu Wang[2], Jialin Meng[2], Qingqing Sun[2], David Wei Zhang[2], and Lin Chen[2,3*]

[1] School of Integrated Circuits, Anhui University, Anhui 230601, P. R. China

[2] School of Microelectronics, Fudan University, Shanghai 200433, P. R. China

[3] Zhangjiang Fudan International Innovation Center, Shanghai 201203, China

[#] These authors contributed equally to this work.

[*]Correspondence and requests for materials should be addressed to Qingxuan Li (liqx@ahu.edu.cn), and Lin Chen (linchen@fudan.edu.cn).

This PDF file includes:

Supplementary Figures 1 to 17 (Pages S4-S12)

Supplementary Tables 1 to 3 (Pages S13-S15)

Supplementary References (Page S16-S17)

Supplementary Fig.1 The micrograph of ITO FeFET.

Supplementary Fig.2 Cross-sectional TEM and EDS images of FeFETs with ultra-thin 4nm ITO channel.

Supplementary Fig.3 The CasaXPS software usage diagram.

Supplementary Fig.4 XPS spectra of ITO film and HZO film.

Supplementary Fig.5 Atomic force microscope (AFM) analysis of the surface morphology of HZO film.

Supplementary Fig.6 The leakage current of the HZO gate stack and the transistor gate current.

Supplementary Fig.7 I_D-V_D curve under a small range of drain voltage.

Supplementary Fig.8 The distribution of key performance indicators (KPIs) for 30 devices.

Supplementary Fig.9 Real-time test photos of the ITO FeFETs devices.

Supplementary Fig.10 The performance of the HZO gate stack and FeFETs under different bending radii.

Supplementary Fig.11 The performance of the HZO gate stack and FeFETs under different bending cycles.

Supplementary Fig.12 Schematic diagram of ANN in the MNIST pattern recognition process.

Supplementary Fig.13 Flowchart illustrating the training process of the neural network for digit image recognition.

Supplementary Fig.14 The relationship between network recognition accuracy and

ANN parameters.

Supplementary Fig.15 Relationship between the network's recognition accuracy and the proportion of noise.

Supplementary Fig.16 The average confusion matrix of the training results changes with the number of training epochs.

Supplementary Fig. 17 The finite element analysis model for the device.

Supplementary Table 1. Benchmarks of MW and I_{ON}/I_{OFF} performance of FeFETs reported in this work versus recently reported FeFETs.

Supplementary Table 2. Retention and endurance performance benchmarks of FeFETs reported in this work versus recently reported FeFETs.

Supplementary Table 3. The parameters for modeling materials in the finite element analysis

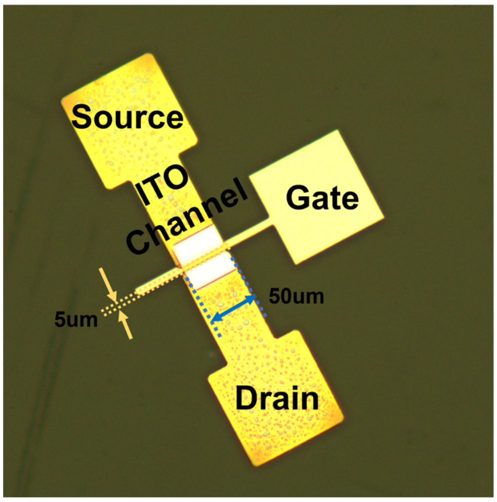

Supplementary Fig.1 The micrograph of ITO FeFET.

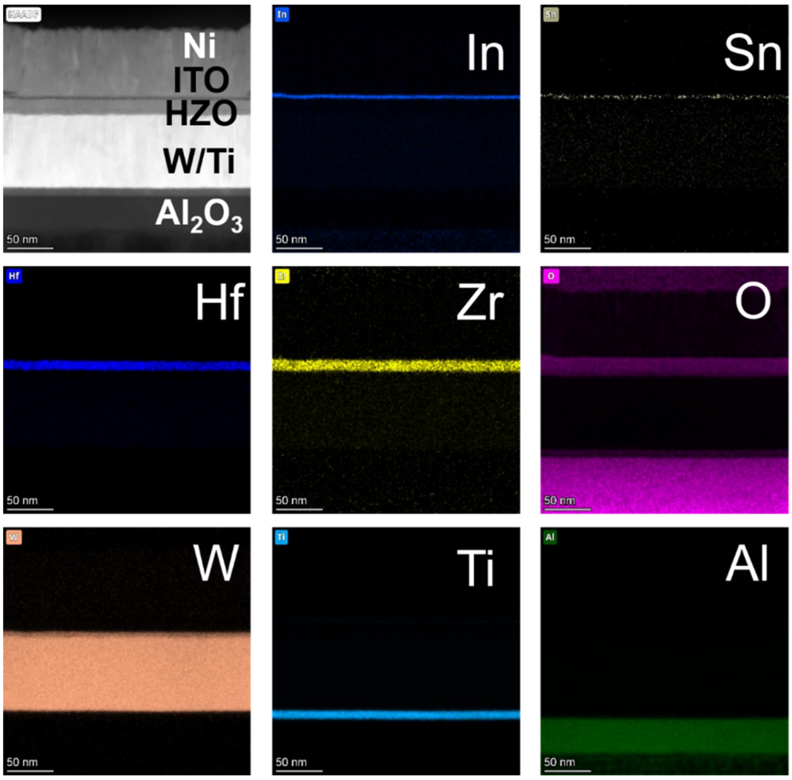

Supplementary Fig.2 Cross-sectional TEM and EDS images of FeFETs with ultra-thin 3.4 nm ITO channel.

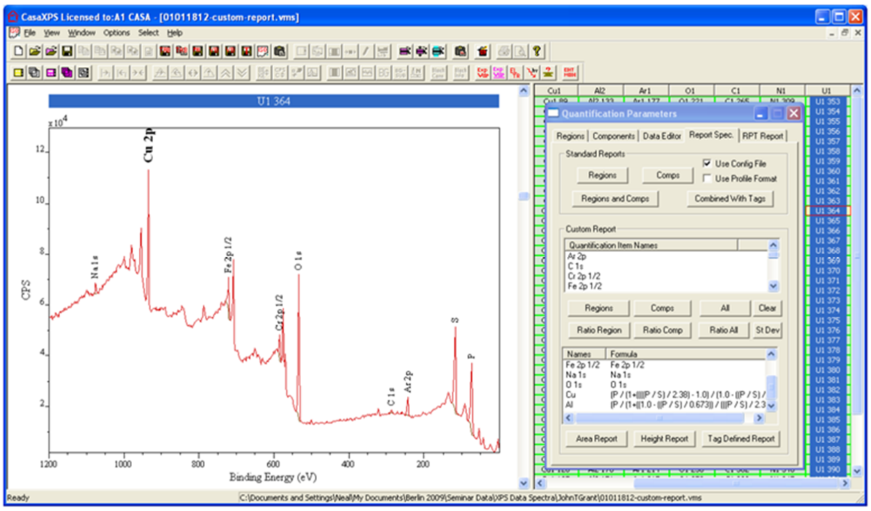

Supplementary Fig. 3 The CasaXPS software usage diagram[1].

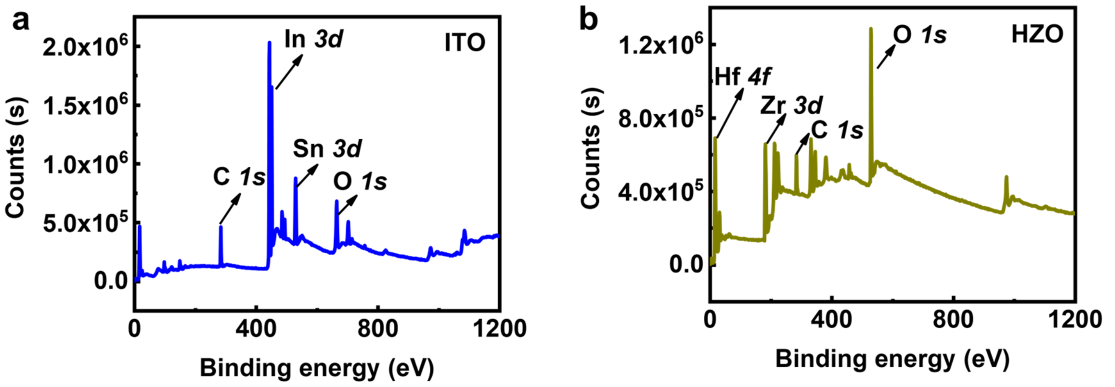

Supplementary Fig.4 XPS spectra of ITO film and HZO film. (a) XPS spectrum of ITO film, including counts of C*1S*, In *3d*, Sn *3d* and O *1s*. (b) XPS spectrum of HZO film, including counts of Hf *3f*, Zr *3d*, C *1s* and O *1s*.

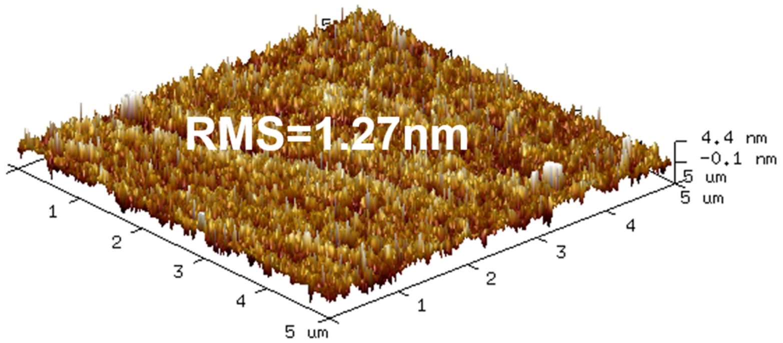

Supplementary Fig.5 Atomic force microscope (AFM) analysis of the surface morphology of HZO film, the root mean square (RMS) roughness is 1.27 nm.

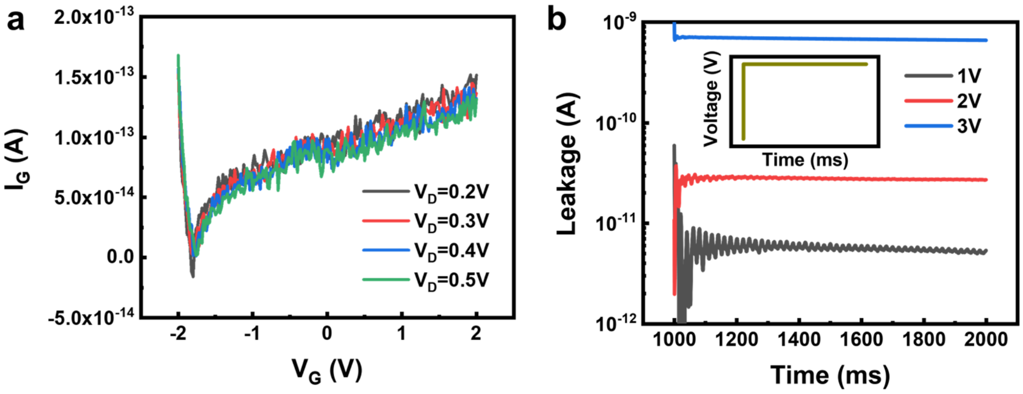

Supplementary Fig.6 The leakage current of the HZO gate stack and the transistor gate current. (a) Measured I_G-V_G curve, showing sub-pA. gate/substrate leakage. (b) Leakage current of the HZO gate stack under different applied voltages.

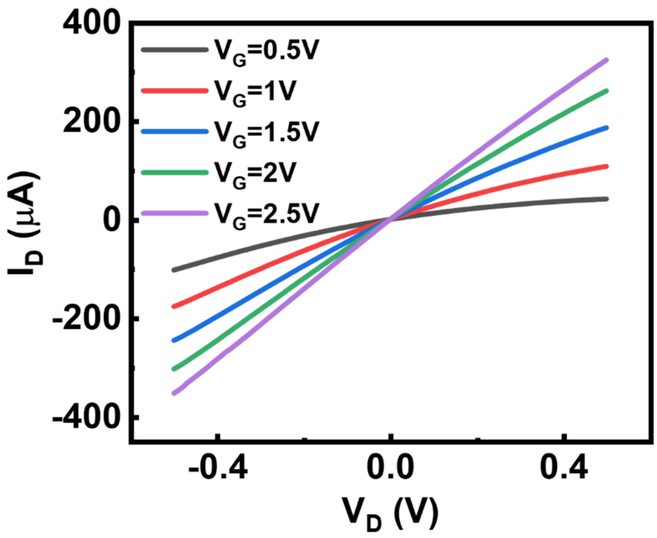

Supplementary Fig.7 I_D-V_D curve under a small range of drain voltage.

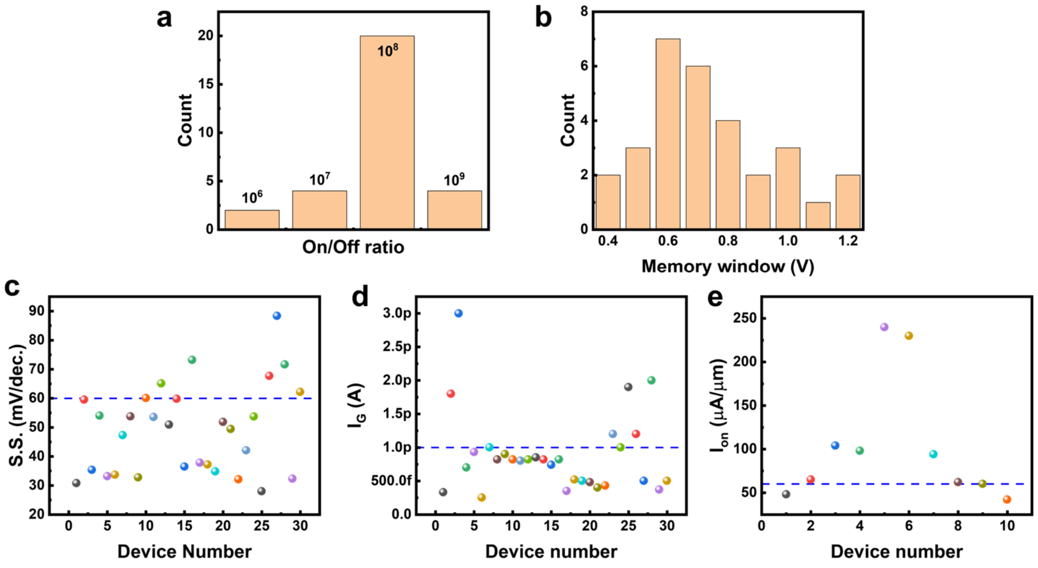

Supplementary Fig.8 The distribution of key performance indicators (KPIs) for 30 devices. Distribution of (a) on/off ratio and (b) memory window for the devices. Statistics of (c) subthreshold swing (S.S.) and (d) gate leakage current (I_G). (e) Statistics of on-state current (I_{ON}).

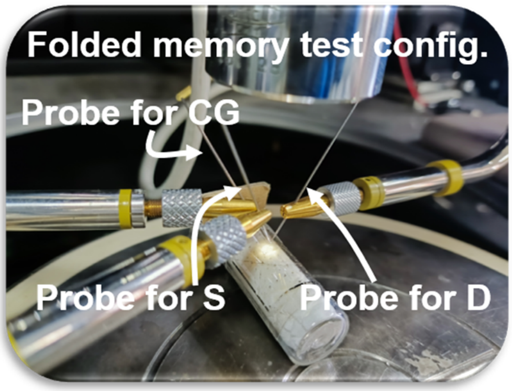

Supplementary Fig.9 Real-time test photos of the ITO FeFETs devices on the ultrathin MICA being characterized under the folded state.

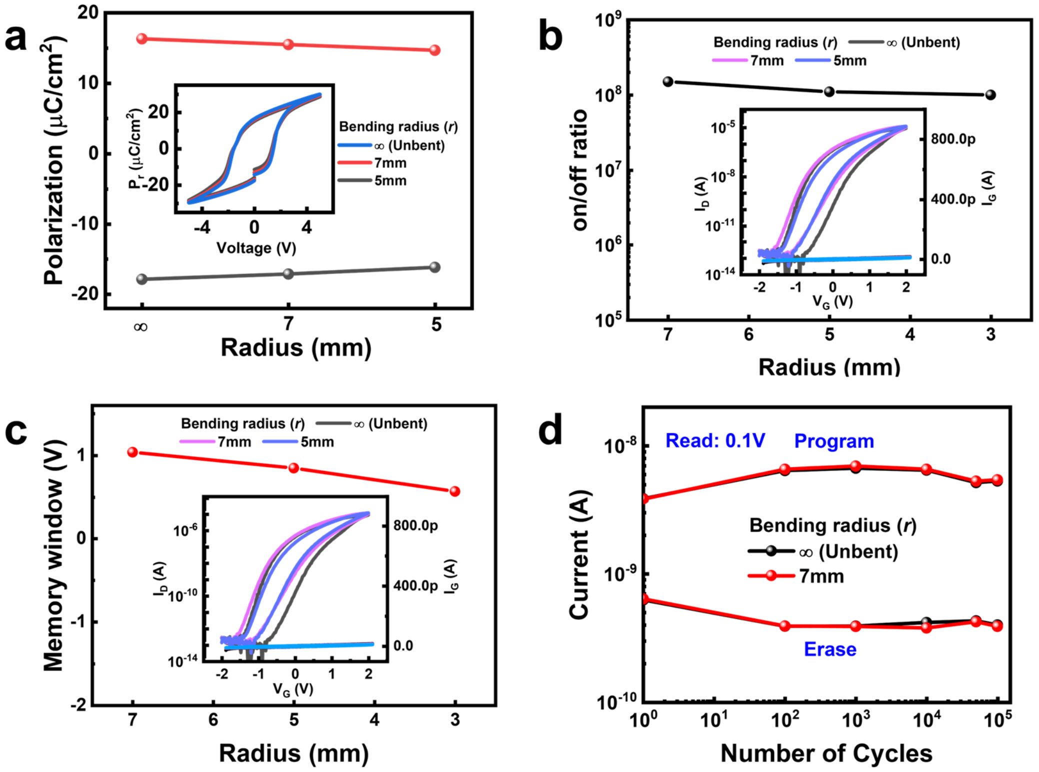

Supplementary Fig.10 The performance of the HZO gate stack and FeFETs under different bending radii. (a) P-V hysteresis loops measured under various bending radii. (b) On/off current ratio and (c) memory window measured in FeFET as a function of the bending radius. These measurements were performed at $V_D = 0.1$ V. (d) Performance

of the endurance property for the ITO FeFETs with different bend radii.

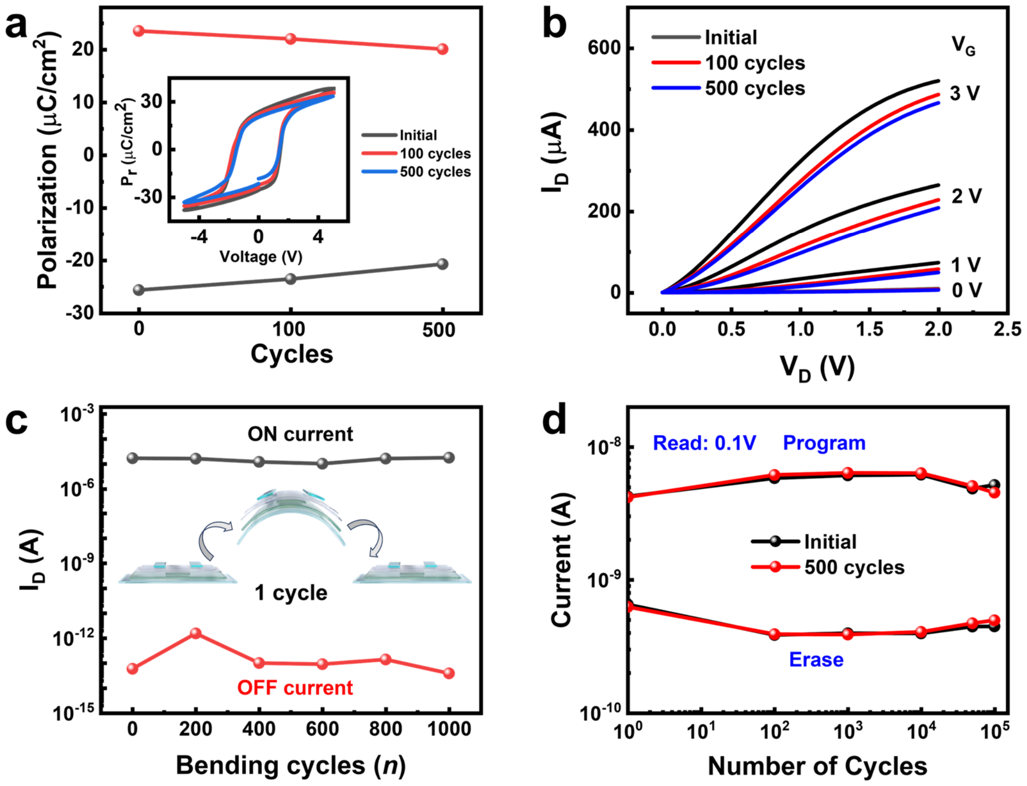

Supplementary Fig.11 The performance of the HZO gate stack and FeFETs under different bending cycles. (a) P-V hysteresis loops measured under various bending cycles. (b) I_D–V_D curve under different gate voltage recorded after various bending cycles. (c) On/off current measured in FeFET as a function of the number of bending cycles. These measurements were performed at $V_D = 0.1$ V with a bending radius of 7 mm. (d) Performance of the endurance property for the ITO FeFETs with different bend cycles.

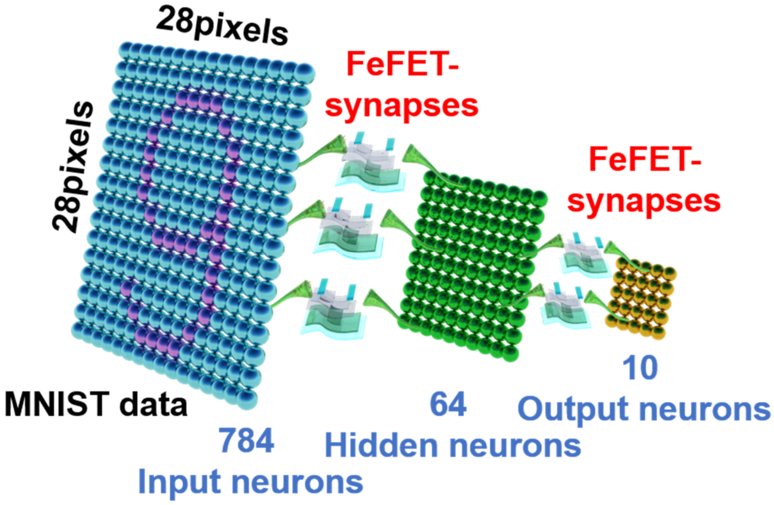

Supplementary Fig.12 Schematic diagram of ANN in the MNIST pattern recognition process.

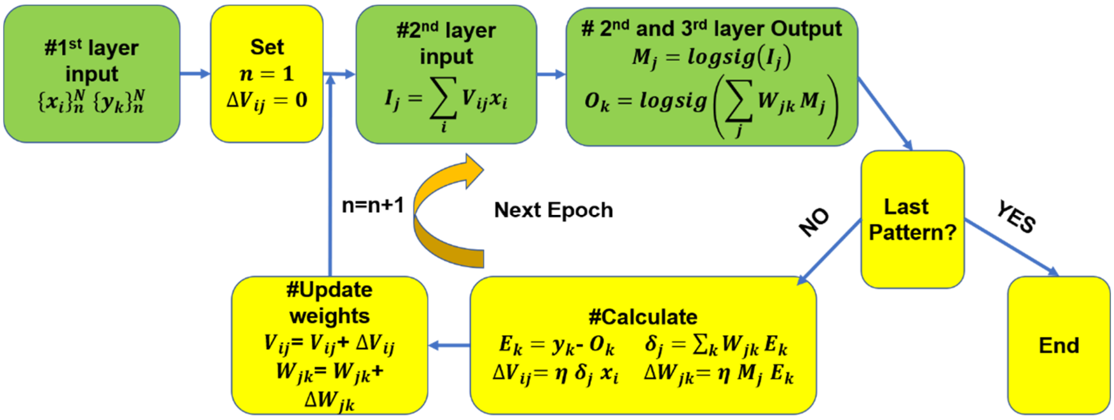

Supplementary Fig.13 Flowchart illustrating the training process of the neural network for digit image recognition.

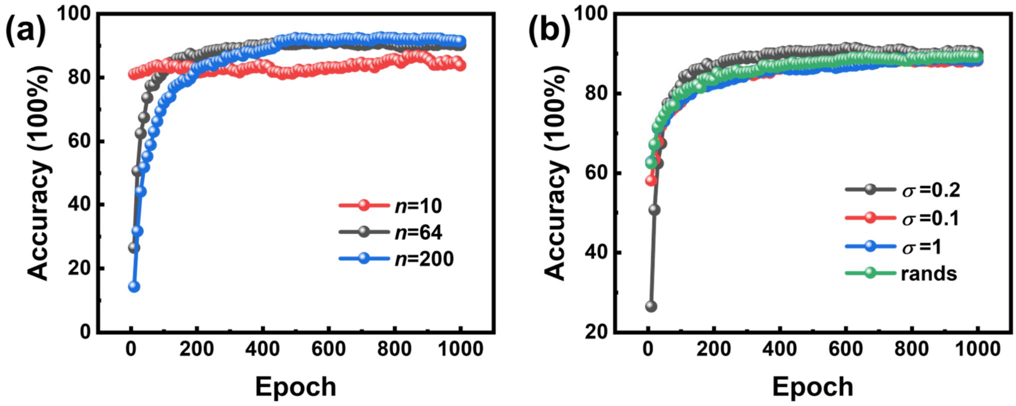

Supplementary Fig.14 The relationship between network recognition accuracy and ANN parameters. (a) Impact of the number of neurons in the hidden layer on the network's recognition accuracy. (b) Effect of the initial values of V and W on recognition accuracy.

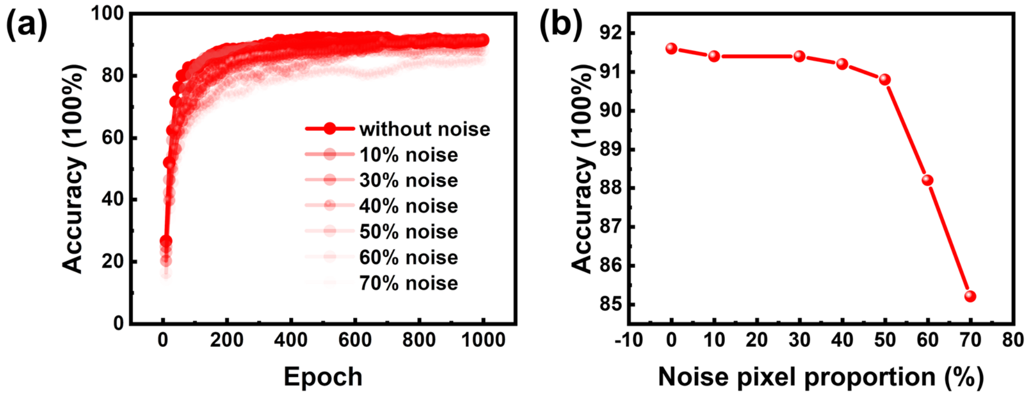

Supplementary Fig.15 The fault tolerance of constructed ANN. (a) Relationship between the network's recognition accuracy and the proportion of noise. (b) With increasing noise pixel ratios, the network's recognition accuracy gradually declined.

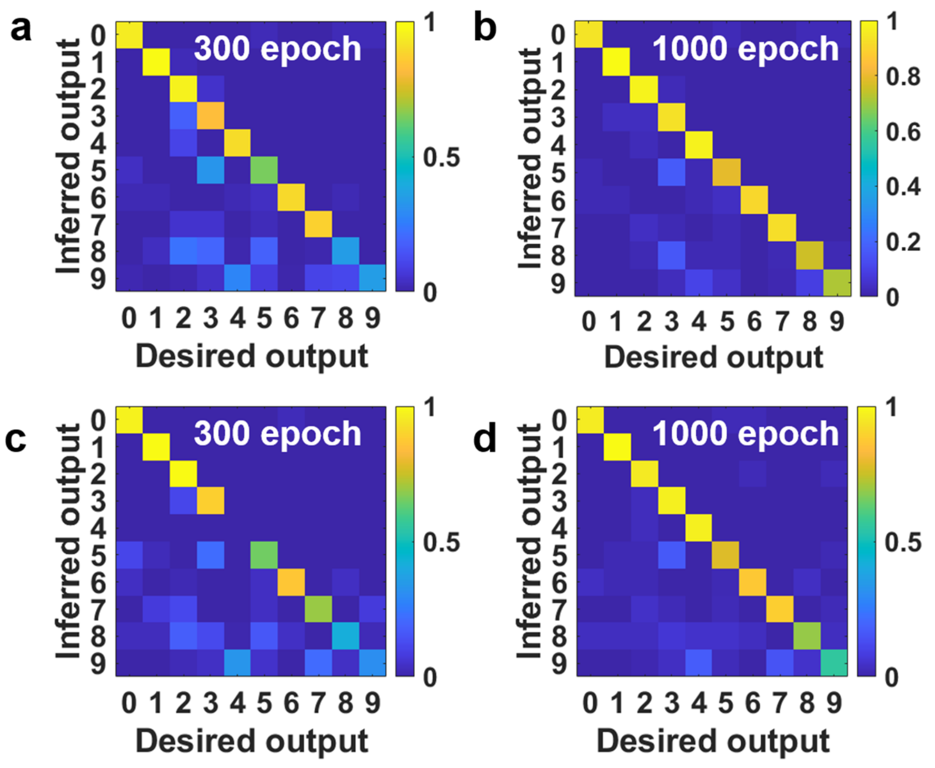

Supplementary Fig. 16 The average confusion matrix of the training results changes with the number of training epochs. Images after (a-b) 600 pulses and (c-d) 48,000 pulses.

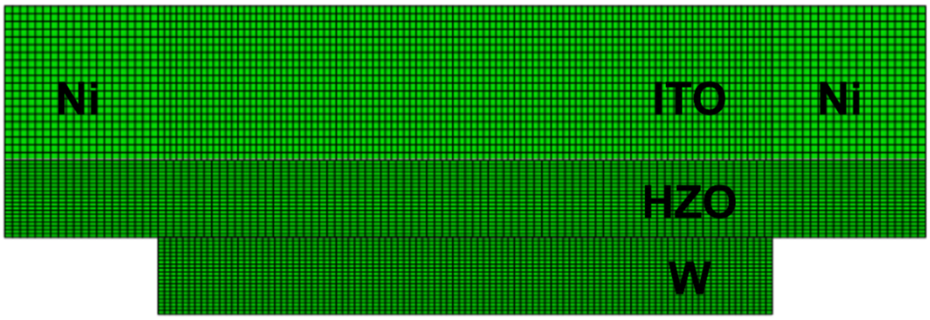

Supplementary Fig. 17 The finite element analysis model for the device. A two-dimensional model was constructed based on the consideration of the device's thin-film stacking structure. The model assumes perfect bonding at the interfaces between different layers.

Supplementary Table 1. Benchmarks of MW and I_{ON}/I_{OFF} performance of FeFETs reported in this work versus recently reported FeFETs, where the MW is normalized with respect to the ferroelectric layer thickness.

**Channel**	**FE**	**I_{ON}/I_{OFF}**	**S.S. (mV/dec.)**	**MW (V/nm)**	**year**	**Ref.**
Poly-Si	HfSiO	10^5	N.A.	0.12	2020	2
IGZO	HZO	10^6	62	0.11	2020	3
a-IGZO	HZO	10^6	N.A.	0.18	2021	4
β-Ga_2O_3	α-In_2Se_3	10^6	72	0.024	2021	5
IZTO	HZO	10^5	N.A.	0.0625	2021	6
IWO	HZO	10^5	N.A.	0.145	2021	7
In_2O_3	HZO	10^7	40	0.22	2021	8
InO_x	ZrO_2-HZO-ZrO_2	10^3	N.A.	0.08	2022	9
InAs	HZO	10^4	113	0.125	2022	10
a-IGZO	HZO	10^5	125	0.05	2022	11
IWO	HZO	10^5	N.A.	0.2	2022	12
ITO/IGZO	HZO	10^7	62	0.21	2022	13
ITO/IGZO	HZO	10^6	68	0.2	2023	14
α-IGZO	HZO	10^6	N.A.	0.17	2023	15
InSe	HfO_2	10^6	N.A.	0.13	2023	16
$InZnO_x$	HZO	10^5	N.A.	0.16	2023	17
In_2O_3	HZO	10^5	N.A.	0.12	2023	18
MoS_2	AlScN	10^7	N.A.	0.17	2023	19
**ITO**	**HZO**	**10^8**	**33**	**0.24**	**2023**	**This work**

Supplementary Table 2. Retention and endurance performance benchmarks of FeFETs reported in this work versus recently reported FeFETs.

**Channel**	**FE**	**Endurance (#)**	**Retention (s)**	**year**	**Ref.**
Poly-Si	HfSiO	10^5	10^5	2020	2
a-IGZO	HZO	10^5	10^4	2021	4
ITO	HfO_2	10^4	10^4	2021	20
InO_x	ZrO_2-HZO-ZrO_2	10^4	10^3	2022	9
InAs	HZO	10^4	10^3	2022	10
ITO/IGZO	HZO	10^7	10yrs	2022	13
ITO	ScAlN	10^5	10^5	2023	21
ITO/IGZO	HZO	10^7	10^4	2023	14
α-IGZO	HZO	10^4	10^4	2023	15
InSe	HfO_2	10^2	10^4	2023	16
$InZnO_x$	HZO	10^6	10^5	2023	17
In_2O_3	HZO	10^3	10^4	2023	18
MoS_2	AlScN	10^4	10yrs	2023	19
**ITO**	**HZO**	**>2x10^7**	**10yrs**	**2023**	**This work**

Supplementary Table 3. The parameters for modeling materials in the finite element analysis. This includes the elastic modulus and Poisson's ratio for ITO and HZO thin films, as well as the elastic modulus and Poisson's ratio for Ni and W electrodes.

	**Poisson's ratio (GPa)**	**Elastic modulus**
Ni	190	0.33
ITO	100	0.33
HZO	150	0.25
W	400	0.31

References

1 http://www.casaxps.com/help_manual/ from CasaXPS, 2013.

2 Saitoh, M. *et al.* in *2020 IEEE International Electron Devices Meeting (IEDM).* 18.11. 11-18.11. 14 (IEEE).

3 Mo, F. *et al.* Low-voltage operating ferroelectric FET with ultrathin IGZO channel for high-density memory application. *IEEE Journal of the Electron Devices Society* **8**, 717-723 (2020).

4 Sun, C. *et al.* Temperature-dependent operation of InGaZnO ferroelectric thin-film transistors with a metal-ferroelectric-metal-insulator-semiconductor structure. *IEEE Electron Device Letters* **42**, 1786-1789 (2021).

5 Yang, J. Y., Yeom, M. J., Park, Y., Heo, J. & Yoo, G. Ferroelectric α-In2Se3 Wrapped-Gate β-Ga2O3 Field-Effect Transistors for Dynamic Threshold Voltage Control. *Advanced Electronic Materials* **7**, 2100306 (2021).

6 Kim, M.-K., Kim, I.-J. & Lee, J.-S. Oxide semiconductor-based ferroelectric thin-film transistors for advanced neuromorphic computing. *Applied Physics Letters* **118** (2021).

7 Aabrar, K. A. *et al.* in *2021 IEEE International Electron Devices Meeting (IEDM).* 19.16. 11-19.16. 14 (IEEE).

8 Lin, Z. *et al.* in *2021 IEEE International Electron Devices Meeting (IEDM).* 17.14. 11-17.14. 14 (IEEE).

9 Li, Z. *et al.* A 3D vertical-channel ferroelectric/anti-ferroelectric FET with indium oxide. *IEEE Electron Device Letters* **43**, 1227-1230 (2022).

10 Persson, A. E., Zhu, Z., Athle, R. & Wernersson, L.-E. Integration of ferroelectric Hf x Zr 1-x O 2 on vertical III-V nanowire gate-all-around FETs on silicon. *IEEE Electron Device Letters* **43**, 854-857 (2022).

11 Hasan, M. M. *et al.* Improvement of Amorphous InGaZnO Thin-Film Transistor With Ferroelectric ZrO x/HfZrO Gate Insulator by 2 Step Sequential Ar/O 2 Treatment. *IEEE Electron Device Letters* **43**, 725-728 (2022).

12 Aabrar, K. A. *et al.* BEOL-compatible superlattice FEFET analog synapse with improved linearity and symmetry of weight update. *IEEE Transactions on Electron Devices* **69**, 2094-2100 (2022).

13 Chen, C.-K. *et al.* in *2022 International Electron Devices Meeting (IEDM).* 6.1. 1-6.1. 4 (IEEE).

14 Chen, C.-K. *et al.* High-Performance Top-Gated and Double-Gated Oxide–Semiconductor Ferroelectric Field-Effect Transistor Enabled by Channel Defect Self-Compensation Effect. *IEEE Transactions on Electron Devices* **70**, 2098-2105 (2023).

15 Jeong, S. *et al.* All-Sputter-Deposited Hf 0.5 Zr 0.5 O 2 Double-Gate Ferroelectric Thin-Film Transistor with Amorphous Indium–Gallium–Zinc Oxide Channel. *IEEE Electron Device Letters* (2023).

16 Liao, J. *et al.* Van der Waals Ferroelectric Semiconductor Field Effect Transistor for In-Memory Computing. *ACS nano* **17**, 6095-6102 (2023).

17 Kim, I.-J., Kim, M.-K. & Lee, J.-S. Design Strategy to Improve Memory Window in Ferroelectric Transistors With Oxide Semiconductor Channel. *IEEE Electron Device Letters* **44**, 249-252 (2022).

18 Luo, C. *et al.* Ferroelectric Hf0. 5Zr0. 5O2-gated synaptic transistors with large conductance dynamic range and multilevel states. *Science China Materials*, 1-11 (2023).

19 Kim, K.-H. *et al.* Scalable CMOS back-end-of-line-compatible AlScN/two-dimensional channel ferroelectric field-effect transistors. *Nat. Nanotechnol.*, 1-7 (2023).

20 Choi, S.-N., Moon, S.-E. & Yoon, S.-M. Impact of oxide gate electrode for ferroelectric field-effect transistors with metal-ferroelectric-metal-insulator-semiconductor gate stack using undoped HfO2 thin films prepared by atomic layer deposition. *Nanotechnology* **32**, 085709 (2020).

21 Mondal, S. *et al.* ScAlN-Based ITO Channel Ferroelectric Field-Effect Transistors With Large Memory Window. *IEEE Transactions on Electron Devices* (2023).
